# Prevalence, Incidence Density, and Genotype Distribution of GB Virus C Infection in a Cohort of Recently HIV-1-Infected Subjects in Sao Paulo, Brazil

**DOI:** 10.1371/journal.pone.0018407

**Published:** 2011-04-05

**Authors:** Maria Teresa M. Giret, João Luiz Miraglia, Maria Cecília Araripe Sucupira, Anna Nishiya, José Eduardo Levi, Ricardo S. Diaz, Ester C. Sabino, Esper G. Kallas

**Affiliations:** 1 Division of Clinical Immunology and Allergy, University of Sao Paulo, Sao Paulo, Brazil; 2 Infectious Diseases Division, Federal University of Sao Paulo, Sao Paulo, Brazil; 3 Sao Paulo Blood Bank, Sao Paulo, Brazil; 4 Institute of Tropical Medicine, University of Sao Paulo, Sao Paulo, Brazil; University of Oxford, United Kingdom

## Abstract

**Background:**

The results of previous studies elsewhere have indicated that GB virus C (GBV-C) infection is frequent in patients infected with the human immunodeficiency virus type 1 (HIV-1) due to similar transmission routes of both viruses. The aim of this study was to determine the prevalence, incidence density and genotypic characteristics of GBV-C in this population.

**Methodology/Principal Findings:**

The study population included 233 patients from a cohort primarily comprised of homosexual men recently infected with HIV-1 in São Paulo, Brazil. The presence of GBV-C RNA was determined in plasma samples by reverse transcriptase-nested polymerase chain reaction and quantified by real-time PCR. GBV-C genotypes were determined by direct sequencing. HIV viral load, CD4+ T lymphocyte and CD8+ T lymphocyte count were also tested in all patients. The overall prevalence of GBV-C infection was 0.23 (95% CI: 0.18 to 0.29) in the study group. There was no significant difference between patients with and without GBV-C infection and Glycoprotein E2 antibody presence regarding age, sex, HIV-1 viral load, CD4+ and CD8+T cell counts and treatment with antiretroviral drugs. An inverse correlation was observed between GBV-C and HIV-1 loads at enrollment and after one year. Also, a positive but not significant correlation was observed between GBV-C load and CD4+ T lymphocyte. Phylogenetic analysis of the GBV-C isolates revealed the presence of genotype 1 and genotype 2, these sub classified into subtype 2a and 2b.

**Conclusion/Significance:**

GBV-C infection is common in recently HIV -1 infected patients in Sao Paulo, Brazil and the predominant genotype is 2b. This study provides the first report of the GBV-C prevalence at the time of diagnosis of HIV-1 and the incidence density of GBV-C infection in one year.

## Introduction

The GB virus type C (GBV-C, also known as hepatitis G virus) is an enveloped, positive-sense, single- stranded RNA virus belonging to the family *Flaviviridae*, and is closely related to the hepatitis C virus (HCV). It has been proposed to be classified as members of a fourth genus in the *Flaviviridae* family, named Pegivirus [1]. GBV-C appears to be lymphotropic and has been shown to replicate *in vitro* in peripheral blood mononuclear cells CD4+ and CD8+ T lymphocytes and B lymphocytes [2], [3]. It was first identified in 1995 in serum from individuals with idiopathic hepatitis [4], [5], [6], [7]. Although infection with GBV-C is common in the HIV-1 infected population, it has not been associated with chronic disease or affect the clinical course of hepatitis A, B, or C infection(s) [8]. Over the past several years a number of studies have found GBV-C to exert a favorable impact on the course of HIV-1 [9], [10], [11], [12], [13] or HCV infections [14], [15] with a lower mortality rate, slower progression to AIDS, and longer survival once AIDS has developed. Others studies have failed to demonstrate a similar effect [16], [17], [18], [19]. Likewise, the discrepancy between results of some studies could be explained by different stage of HIV-1 infection among the different population studied [20].

GBV-C is prevalent among subjects highly exposed to HIV-1 acquisition and those with asymptomatic or symptomatic HIV infection [21], as it is probably sexually transmitted. It is also known as having transmitted via blood, blood products, intravenous drug use, and from mother to child through pregnancy and/or delivery [22], [23].

The geographical distribution is related to the co-evolution of the viruses with humans during the migrations along the history, suggesting that GBV-C is an ancient virus [24], [25]. The phylogenetics analyses of GBV-C isolates have demonstrated the presence of multiple genotypes with consistent geographical clustering. Moreover, there is a high degree of nucleotide and amino acid sequence conservation between isolates from widely separated geographic areas [26]. Genotype 1 is found in West Africa [27]; genotype 2 (sub-classified as either 2a or 2b) in the United States and Europe [26]; genotype 3 in Asia [28], [29], [30]; genotype 4 in Myanmar and Vietnam [31]; genotype 5 in South Africa [32]; and genotype 6 in [33], [34]. A geographic sub-cluster within the GBV-C genotype 2 has been recently identified in Portugal [35].

The frequency of GBV-C infection in patients with HIV-1 ranges from as low as 13.5% [36] in a Argentinean population of HCV+/HIV+ hemophilic patients, from 24% to 37% in a group comprised predominantly by male homosexuals in the United States [37], in a similar Danish cohort [37], [38], [39] in a Brazilian group of heterosexual HIV-1 infected participants [37], [38], [39] and 45% in France, in a cohort of HIV-1 infected patients where intravenous drug use and homosexuality were identified as major transmission risk factors [40]. The frequency of GBV-C exposure in children with chronic renal failure has been shown to be high as 51% comparing to the healthy group with 8%, and may be the result of frequent blood transfusions [41]. Moreover, the GBV-C genotypes were not analyzed. Ramezani *et al.* studying 82 Iranian HIV positive patients found a GBV-C prevalence of 10.97% and there was no statistically significant difference in the prevalence between the two groups studied: 13.5% in IDUs (13.5%) vs. heterosexuals (6.7%)[42]. The results of this study indicate that the parental route of transmission may be an important method of viral transmission, though other routes ie sexual [43] and household contact [44] may also play a role in GBV-C epidemiology. The prevalence of GBV-C infection was 9% in a group of 64 hemodialysis patients from Caracas, Venezuela [45], 13.6%, all genotype 2, in a total of 104 hemodialysis patients living in Tehran [46]. In an Amerindian population from Venezuela a high prevalence of GBV-C genotype 3 was observed, ranging from 5% (9 out of 162) in the West to 25% (14 out of 56) in the South region of the country [45]. The prevalence of GBV-C was 9.7% among 545 blood donors in Sao Paulo, Brazil [47], 8.3% in 1,039 healthy individuals, also from São Paulo, Brazil [48] or 1% from 478 Iranian volunteer blood donors [49].

Following its discovery and description; several studies began reporting GBV-C virus prevalence in HIV-1 uninfected patients from different groups and regions in Brazil. First reports were initially available from hemodialysis patients with prevalence of 15%, or from patients with chronic liver diseases with a prevalence of 19% and 7% respectively [50], [51], [52]. The spread of GBV-C infection is also described in the general population ranging from 2% to 18% and the predominant genotypes were 2a, 2b and 1 [47], [53], [54], [55], [56], [57], [58].

Since the available data on the GBV-C epidemiology among Brazilian HIV-1-infected patients is limited to cross-sectional studies, the aim of this work was to determine the prevalence, incidence density, and genotypic distribution of GBV-C in a cohort of recently HIV-1-infected subjects in Sao Paulo, Brazil.

## Methods

### Ethics Statement

Informed written consent was obtained from all the patients and the study was approved by the Ethics Committees and the Institutional Review Board of the Federal University of Sao Paulo [#0919/01].

### Study sample

Subjects included in this study were individuals seeking HIV testing at counseling and testing centers located in Sao Paulo, Brazil. Recent HIV-1-infection was determined by the Serologic Testing Algorithm for Recent HIV Seroconversion (STARHS), and individuals were included in the study when they had a negative desensitized ELISA HIV-test, that could indicate an incomplete antibody response as a consequence of recent HIV-1 infection [59], [60].

The volunteers were evaluated for clinical and virological status, as well as, for the presence of other co-infections at enrollment [61] and after one year of follow up. Plasma and peripheral blood mononuclear cells (PBMC) were obtained, the latter from leukapheresis with Ficoll-Paque (Pharmacia Biotech, Uppsala, Sweden) density gradient centrifugation. The age, gender, race, HIV transmission route, and laboratory data are described in Table 1.

**Table 1:** Characteristics and comparisson of the population from Group 1, according to their GBV-C status at baseline, N = 233.

	GBV-C RNA Positive n = 54	GBV-C RNA Negative n = 179	*p*
**Gender (n,%)**			
Male	50 (92.6)	162 (90.5)	0.63*
**Ethnicity (n,%)**			
White	29 (53.7)	103 (60.23)	0.69*
Mulato	11 (20.37)	34 (19.88)	
Black	4 (7.41)	13 (7.6)	
Other	10 (18.52)	21 (12.28)	
**Exposure (n,%)**			
MSM	45 (83.33)	146 (82.95)	0.97*
Hetero	9 (16.67)	30 (17.05)	
**Age, in years**			
**median (IQR 25-75)**	27.61 (24–35)	31.48 (26–38)	0.018**
**CD4+ Tcells, in cells/mm^3^**			
**median (IQR 25–75)**	512.5 (401–604)	521 (398–709)	0.99**
**CD8+ Tcells, in cells/mm^3^**			
**median (IQR 25–75)**	940 (641–1,151)	877 (601–1,243)	0.76**
**HIV RNA (log copies/mL)**			
**median (IQR 25–75)**	4.30 (3.60–4.91)	4.29 (3.59–4.81)	0.87**
**GBV-C RNA (log au/mL)**			
**median (IQR 25–75)**	3.06 (2.44–4.44)		

*Pearson's chi-squared test.

**Wilcoxon rank-sum test adjusted for ties.

The initiation of antiretroviral therapy followed the Ministry of Health Guidelines for HIV therapy in Brazil [62] which recommends treatment for patients with CD4+ T cell counts bellow 200 cells/µL, as well as, to consider treatment for patients with CD4+ T cell counts between 200 and 350 cells/µL, or in the presence of clinical AIDS. As the standard clinical practice in this cohort, treatment was initiated when CD4+ T cell counts were confirmed bellow 300 cells/µL.

### CD4+ T cell counts and Viral Load (VL) quantification

The CD4+ and CD8+ T cell counts were measured by using lymphocyte staining with CD3, CD4, and CD8 conjugated monoclonal antibodies (TriTest, BD Biosciences, San Diego, California, USA). HIV plasma viral load was determined using the Amplicor HIV-1 Monitor test, version 1.5 (Roche Diagnostics Systems, Branchburg, New Jersey, USA) with a detection limit of 400 copies/ml until January 2007. This test was subsequently replaced by the branched DNA assay (Versant® HIV-1 RNA 3.0 ASSAY (bDNA), Siemens Healthcare Diagnostics, Deerfield, IL, USA) with a detection limit of 50 copies/ml.

At baseline (visit 1), upon the confirmation of recent HIV-1-infection, all included volunteers, to be referred to as Group 1, had a plasma sample tested for the presence of GBV-C RNA, and were classified as having active GBV-C infection (GBV-C RNA present) or no GBV-C infection (GBV-C RNA absent). All volunteers from Group 1 with active GBV-C infection, also had their GBV-C viral load, and GBV-C genotype determined at visit 1.

From the total of 233 volunteers belonging to Group 1, a subgroup of 131 subjects, referred as Group 2, was also tested at visit 1 for the presence of E2 antibodies against GBV-C envelope glycoprotein E2 (anti-E2), and was classified as having active GBV-C infection (GBV-C RNA present, and anti-E2 absent), past GBV-C infection (GBV-C RNA absent, and anti-E2 present), and susceptible to GBV-C infection (GBV-C RNA absent, and anti-E2 absent). These volunteers were also tested for the presence of GBV-C RNA and E2 antibody after one year of the enrollment (visit 5), after the confirmation of recent HIV-1-infection. They were reclassified at visit 5 as cases of cleared GBV-C infection (GBV-C RNA absent), or new cases of active GBV-C infection (GBV-C RNA present), respectively.

### Detection of E2 antibody

As markers of GBV-C RNA clearance and prior exposure [63] plasma E2 antibodies were detected using an immunoassay using recombinant E2 (mPlate Anti-Hgenv test; Roche Diagnostics, kindly provided by Dietmar Zdunek), in accordance with the manufacturer's instructions. Plates were incubated with diluted (1:20) serum, and E2 antibodies were detected using anti-human immunoglobulin G peroxidase conjugate and 2,2′-azinobis (3-ethylbenzthiazoline-6-sulfonic acid) (ABTS) substrate. In accordance with the manufacturer's cutoff, an optical density (OD) of less than 0.10 was considered to be negative, and an OD of at least 0.10 was considered to be positive.

### Detection and quantification of GB virus type C RNA

Viral RNA was extracted from 140 µL plasma samples using QIAamp Viral RNA Mini Kit (QIAGEN Inc, CA), according to the manufacturer's instructions. The quantity of 5 µL of the RNA extracted was diluted in a mix containing 150 ng of random primer (Random Primer, Pharmacia Biotech, Sweden) and 0.5 mM deoxyribonucleosides triphosphate (dNTPs by Invitrogen Inc., Carlsbad, California, USA); the solution was kept at 65°C for 5 minutes. Complementary DNA (cDNA) synthesis was carried out by the addition of 200 U of Super Script III Reverse transcriptase (Invitrogen Inc, CA) in a buffer solution with 10 U of ribonuclease inhibitor (Invitrogen Inc, CA) at 25°C for 5 minutes, 50°C for 60 minutes and 70°C for 15 minutes at a final volume of 20 µL.

A fragment of 344 bp from the 5′ non-coding region (5′ NCR) was amplified by nested RT PCR using the followings primers located at positions 108 (5′-AGGTGGTGGATGGGTGAT-3′; sense, outer), 134 (5′-TGGTAGGTCGTAAATCCCGGT-3′; sense, inner), 476 (5′-GGAGCTGGGTGGCCCCATGCAT-3′; antisense, inner) and 531 (5′-TGCCACCCGCCCTCACCCGAA-3′; antisense, outer) [32], [64]. Amplification consisted of 40 cycles for both first and second rounds of PCR, with the following incubation times and temperatures: 94°C 30 s, 50°C 30 s, and 72°C 30 s for the first round and 94°C 30 s, 60°C 30 s, and 72°C 30 s for the second round. After amplification, 5 µl of the PCR product was used for electrophoresis analysis on a 2% agarose gel.

The GBV-C load was quantified in all GBV-C RNA-positive samples in triplicate by Real-Time PCR using a TaqMan PCR detection kit (Perkin-Elmer Applied Biosystems, Foster City, California, USA). The following oligonucleotides were used in the real-time PCR located at positions 111–130 (5′-GTGGTGGATGGGTGATGACA-3′; sense), 192-171(5′-GACCCACCTATAGTGGCTACCA-3′, antisense). The GBV-C specific probe tagged with fluorescence FAM CCGGGATTTACGACCTACC NFQ (MGB, Minor Groove Binder; NFQ, nonfluorescent quencher) antisense 154–136 numbered according to Accession NC_001710.1 [65] was synthesized by Applied Biosystems. A strongly positive GBV-C RNA plasma bag from an HIV negative blood donor was obtained and serial dilutions of it were used to estimate the assay endpoint sensitivity. This corresponded to a 10000X dilution of the original plasma. On the basis of that, this standard plasma bag was estimated to contain 10 000 detectable units of GBV-C RNA and was therefore used on real-time assays to quantify viral load in HIV patients. Results are provided in comparison to this “standard”. The limit of detection was one arbitrary unit (au)/mL.

### Sequencing and genotyping

The 344 bp PCR product was purified with the QIAquick kit (QIAGEN, Hilden, Germany), and directly sequenced using Big Dye Terminator Cycle Sequencing Ready Reaction (Applied Biosystems, Foster City, USA) according to the manufacturers protocol. The sequences were edited using the SEQUENCHER program (Gene Codes Corporation Ann Arbor, Michigan, USA) and aligned with the reference sequences of the five main genotypes obtained from GenBank. The phylogenetic analysis was carried out in the 5′ noncoding region (5′ NCR) fragments with the PHYLIP 3.5c program. Bootstrap values were determined on 1000 replicates of the sequence data with the SEQBOOT program. Phylogenetic relationships were constructed using the Neighbor-Joining method, and the pairwise distances were calculated by the maximum likelihood with the DNADIST program. The consensus tree was found with the CONSENSE program. The GBV-C sequences used in this study were deposited in GenBank under accession numbers GQ227302-49.

### Statistical Analysis

The demographic and laboratorial characteristics of volunteers at baseline (visit 1) were compared within Group 1, among the different GBV-C genotypes from the active GBV-C infection cases of Group 1, and within Group 2. The categorical variables were compared by the Pearson's chi-squared test, or by the two-sided Fisher's exact test, while the continuous variables were compared by the Wilcoxon rank-sum test, or the Kruskal-Wallis test, both adjusted for ties when necessary. The *p* values for the Kruskal-Wallis statistic were obtained by the chi-square approximation.

Group 1 was used to calculate the prevalence of active GBV-C infection at visit 1, and Group 2 was used to calculate the prevalence of past GBV-C infection at visit 1, along with their exact binomial 95% confidence intervals. We also calculate the incidence density referred as the total number of cases divided by the total number of person-years of follow-up. The cases of cleared GBV-C infection from Group 1 were used to calculate the incidence density of cleared GBV-C infection at visit 5, and the new cases of active GBV-C infection from Group 2 were used to calculate the incidence density of active GBV-C infection at visit 5, along with their exact Poisson 95% confidence intervals. Linear regression was used to evaluate the impact of GBV-C genotype on GBV-C viral load at visit 1, and included the volunteers with active GBV-C infection from Group 1. The initial full model was adjusted for age (continuous) and gender. The GBV-C viral load between visit 1 and visit 5 was compared, among those volunteers with confirmed GBV-C active infection at both visits, by the Wilcoxon signed rank test.

Two linear regression models were built to evaluate if active GBV-C infection had any impact on CD4+ T cell count, or on HIV viral load at visit 1, and two logistic regression models were built to evaluate if laboratory and demographic characteristics could predict active or past GBV-C infection at visit 1. The volunteers from Group 1 were included in the models evaluating active infection, while the ones from Group 2 were included in the model evaluating past infection. These analyses included in the initial full model age (continuous), gender, ethnicity, and sexual exposure. In addition, the initial linear regression models included CD4+ T cell count at visit 1, when evaluating the impact on HIV viral load, or HIV viral load at visit 1, when evaluating the impact on CD4+ T cell count, while the logistic regression models included both.

The variables included in the final linear regression models were selected by the change-in-estimate procedure with backward elimination [66]. At each stage, the variable for which removal caused the smallest change in the regression coefficient of the exposure of interest was removed, given that this change was smaller than 10%. The backward stepwise regression procedure, with a *p* value of 0.05 for backward-selection, was used to obtain the final models for the logistic regression analysis. All statistical tests used a significance level of 0.05 and were performed using STATA 10.0 (Stata Corporation, Texas).

## Results

### Study Population

A total of 233 antiretroviral naïve subjects were enrolled in the cohort at the moment of the study. One of the major characteristics of the targeted patient population is the majority of men who have sex with men (MSM); other demographic characteristics of the cohort are included In the Table 1. Cross-sectional analyses at time of enrollment (visit 1) and one year after seroconversion (visit 5) were performed. All samples were tested for the presence of GBV-C RNA and a sub-population of 131 patients was tested for the presence of E2 antibody.

### Prevalence and Incidence Density of GB Virus C infection

At enrollment, 54 cases of active GBV-C infection (defined by the presence of GBV-C RNA without E2 antibody) and 31 cases of past GBV-C infection (defined by the presence of E2 antibody without GBV-C RNA) were identified, resulting in a prevalence of 0.23 [95% confidence interval (95% CI), 0.18 to 0.29], and of 0.24 (95% CI: 0.16 to 0.32) respectively. Among the volunteers susceptible to GBV-C infection at visit 1, two new cases of active GBV-C infection were identified after one year (visit 5), resulting in an incidence density (ID) of 3.6 per 100 person-years (95% CI: 0.44 to 13.1). Among the volunteers with active GBV-C infection at visit 1, four cleared the GBV-C viremia at visit 5, resulting in an ID of 10 per 100 person-years (95% CI: 3 to 28). Interestingly, no patient developed E2 antibodies after clearance of GBV-C RNA and also no case of viremia reactivation in antibody positive individuals was observed. Moreover, there was no association between clearance of GBV-C viremia and its genotypes. From those who cleared the virus, only one had begun antiretroviral therapy and among the incident cases, two out of three had initiated antiretroviral therapy after 48 and 204 days of enrollment.

There were no statistically significant differences in the demographic or laboratory characteristics at visit 1 within Group 1 (Table 1) or Group 2 (Table 2) and active GBV-C infection did not have any significant impact on CD4+ T cell count or on HIV-1 viral load.

**Table 2:** Characteristics of the population from Group 2, according to their E2 Antibody status at baseline, N = 131.

	RNA (+) n = 31	E2 (+) n = 31	RNA/E2 (−) n = 69	*p*
**Gender (n,%)**				
Male	28 (90.32)	26 (83.87)	63 (91.3)	0.52*
**Ethnicity (n,%)**				
White	18 (58.06)	21 (67.74)	40 (58.82)	0.12*
Mulato	6 (19.35)	3 (9.68)	17 (25)	
Black	4 (12.9)	7 (22.58)	5 (7.35)	
Other	3 (9.68)		6 (8.82)	
**Exposure**				0.9*
MSM	23 (74.19)	24 (77.42)	54 (78.26)	
Hetero	8 (25.81)	7 (22.58)	15 (21.74)	
**Age, in years**				
**median (IQR 25–75)**	29.07 (24–35)	31.48 (25–36)	32.54 (26–39)	0.099**
**CD4+ Tcells, in cells/mm^3^**				
**median (IQR 25–75)**	534 (389–607)	542 (381–837)	519 (417–727)	0.84**
**CD8+ Tcells, in cells/mm^3^**				
**median (IQR 25–75)**	952 (633–1,121)	952 (637–1,459)	877 (579–1,143)	0.4**
**HIV RNA (log copies/mL)**				
**median (IQR 25–75)**	4.30 (3.54–4.93)	4.39 (3.28–4.69)	4.11 (3.64–4.77)	0.88**

*Pearson's chi-squared test.

**Kruskal-Wallis test (chi-square approximation) adjusted for tiés.

### CD4+ T cell counts and VL quantification

Overall, in the group of GBV-C RNA positive, the median CD4+ T lymphocyte count was 513 cells/mL (interquartile range 25–75% [IQR], 401–604), the median CD8+ T lymphocyte count was 940 cells/mL (IQR 641–1,151), and the median HIV-1 viral load was 19,800 copies/mL (IQR 3,960–80,500) or 4.3 log_10_ copies/mL (IQR 3.60–4.91). In the group of GBV-C RNA negative participants, the median CD4+ T lymphocyte count was 521 cells/mL (IQR 398–709), the median CD8+ T lymphocyte count was 877 cells/mL (IQR 601–1,243), and the median HIV-1 viral load of 19,607 copies/mL (IQR 3,916–63,935), or 4.3 log_10_ copies/mL (IQR 3.60–4.81) (Table 1).

The median viral load of GBV-C at baseline was 1,157 au/mL (IQR 274–27,800), corresponding to 3.06 log_10_ au/mL (IQR 2.44–4.44) (Table 1), and at visit 5 was 11,851 au/mL (IQR 406–62,236), or 4.07 log_10_ au/mL (IQR 2.06–4.79) (*p* value = 0.17).

### Amino Acid Sequence Analyses and Genotype Distribution

**Figure 1:**
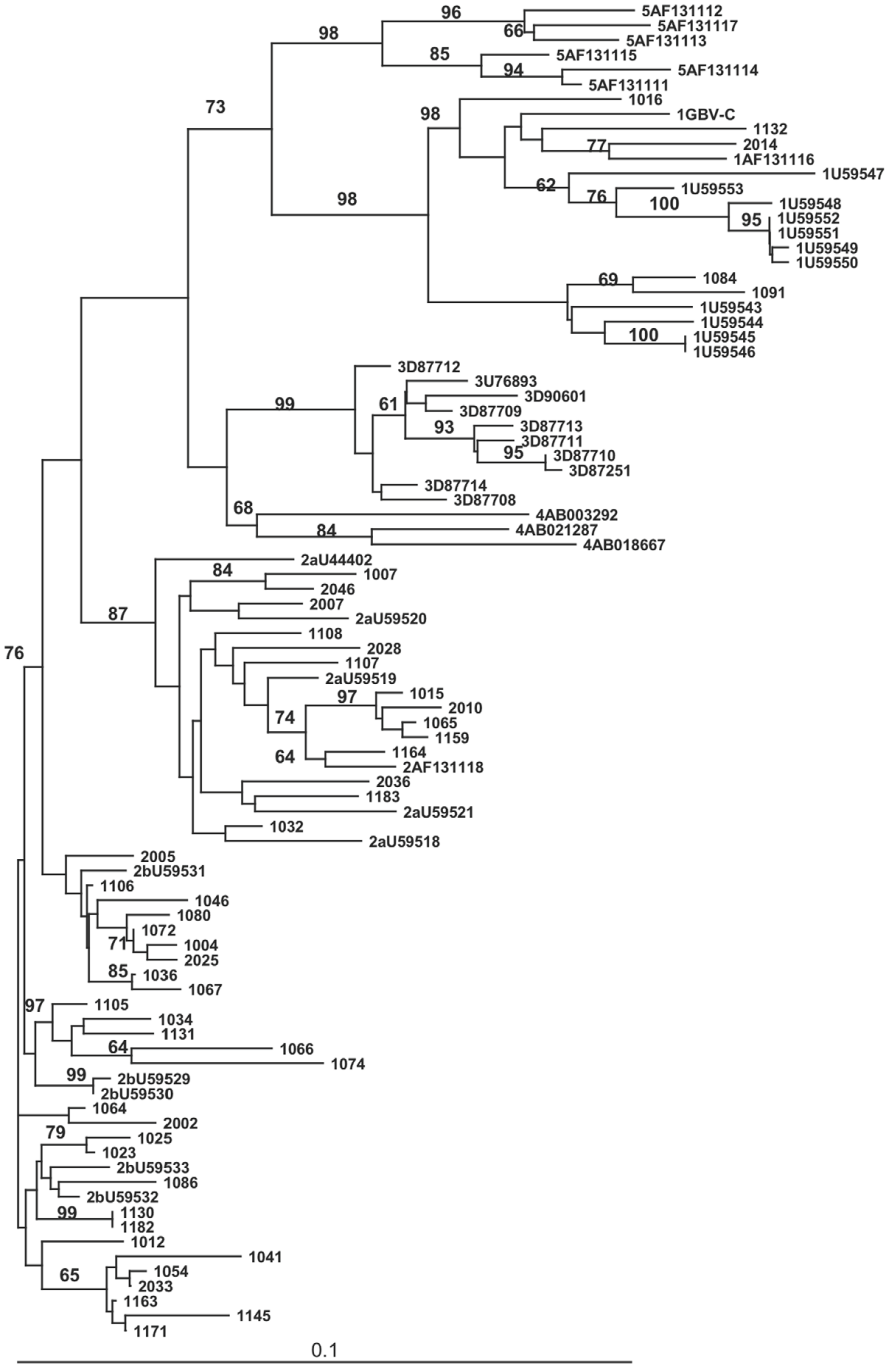
Phylogenetic tree of GB virus C (GBV-C) isolates. Phylogenetic tree of Brazilian GB virus C (GBV-C) clinical isolates based on the nucleotide sequences. Tree was constructed by the Neighbor-Joining (NJ) program, and the pair wise distances between sequences were calculated by the DNADIST program. Values near branches indicate bootstrap support based on 1000 replicates. Relevant bootstrap values >60 (out of 100) are shown.

At visit 1, 45 GBV-C isolates, out of 54 GBV-C RNA-positive samples, could be amplified and the phylogenetic tree revealed that 5 (11%) GBV-C strains were grouped as genotype 1, 13 (28.8%) as genotype 2a, and 27 (60%) as genotype 2b (Figure 1). Among the volunteers from Group 1 with active GBV-C infection, the GBV-C viral load at visit 1 was significantly different (p = 0.007) among genotypes 1, 2a, and 2b (Table 3). The final linear regression model evaluating the impact of GBV-C genotype on GBV-C viral load was adjusted only for age. The mean GBV-C viral load, in the log_10_ scale, of genotype 1 was 2.4 units higher than of genotype 2b (p = 0.001), of genotype 2a was 1 unit higher than of genotype 2b (p = 0.019), and there was no significant difference between genotypes 1 and 2a (p = 0.6).

**Table 3:** Characteristics of the GBV-C positive population, according to their genotypic distribution, N = 45.

	Genotype			
	1 n = 5	2a n = 13	2b n = 27	*p*
**Gender (n,%)**				
Male	5 (100)	13 (100)	23 (85)	0.41*
**Ethnicity (n,%)**				
White	2 (40)	8 (62)	15 (55)	0.94*
Mulato	2 (40)	2 (15)	4 (15)	
Black	0 (0)	1 (8)	3 (11)	
Other	1 (20)	2 (15)	5 (19)	
**Exposure**				0.49*
MSM	4 (80)	12 (92)	20 (74)	
Hetero	1 (20)	1 (8)	7 (26)	
**Age, in years**				
**median (IQR 25–75)**	23.23 (23–24)	25.76 (24–31)	29.08 (26–35)	0.15**
**CD4+ Tcells, in cells/mm^3^**				
**median (IQR 25–75)**	372 (358–546)	590 (403–812)	573 (450–676)	0.38**
**CD8+ Tcells, in cells/mm^3^**				
**median (IQR 25–75)**	752 (649–1,049)	1,002 (763–1,211)	933 (633–1,158)	0.47**
**HIV RNA (log copies/mL)**				
**median (IQR 25–75)**	4.63 (3.6–4.95)	4.45 (3.75–5.10)	3.95 (3.24–4.92)	0.54**
**GBV-C RNA (log au/mL)**				
**median (IQR 25–75)**	5.04 (4.47–5.35)	3.92 (3.00–4.86)	2.70 (2.05–3.07)	0.007**

*Two-sided Fisher's exact test.

**Kruskal-Wallis test (chi-square approximation).

### Relationship between GBV-C infection, HIV-1 viral laod, CD4+ Tcell count and age

The final logistic regression model including volunteers from Group 1 had only age as a statistically significant predictor of active GBV-C infection (p = 0.04). It demonstrated an inverse association of age and active GBV-C infection, which can be translated into a predicted probability of active GBV-C infection of 0.19 among volunteers with 36 years of age (percentile 75), and of 0.27 among volunteers with 25 years of age (percentile 25), a 0.08 change in probability. The final logistic regression model including volunteers from Group 2 did not find any significant association of laboratory or demographic characteristics with past GBV-C infection (data not shown).

We then proceeded with correlation analyses between GBV-C viral load and HIV-1 viral load or CD4+ T cell counts. At the earlier visit, an inverse but not significant correlation (r = −0.24, p = 0.1071) was found, along with no apparent correlation between the GBV-C load and the CD4+ cell count (r = 0.09, p = 0.5069) (data not shown). After one year, when we performed the same analysis excluding those on antiretroviral therapy, a persistent trend of negative correlation between the GBV-C load and the HIV-1 load was documented (r = −0.30, p = 0.1608). At the same time, a trend of positive correlation between the GBV-C load and the CD4+ cell count (r = 0.43, p = 0.07) was observed in the group of untreated patients (data not shown).

## Discussion

In this study we determined the prevalence, incidence density, and genotypic characteristics of GBV-C virus infection in a cohort of 233 patients recently infected with HIV-1 from different counseling and testing centers located in Sao Paulo, Brazil.

This cohort was comprised primarily of ethnically diverse young men predominantly infected by HIV-1 clade B virus who reported having sex with men, and no intravenous drug users [67]. Evidence of GBV-C infection was documented in 47% of participants (23% with ongoing viral replication and 24% with evidence of cleared infection). The prevalence of GBV-C found in our cohort was similar to previous reports in other HIV-1-infected groups from North America [10] and Europe [9], [38], though higher than reported in Argentina [36]. Additionally, our results indicate a higher prevalence than previous studies performed in non HIV-infected Brazilians [47], [53], [54], [56], [68], [69], [70]. The prevalence of GBV-C will likely vary by population but it appears clear that GBV-C infection is more common in groups with risk factors for percutaneous and sexual transmission of infectious agents. The inverse relationship observed between age and active infection may indicate prior infection in older patients who were exposed to GBV-C at a time before HIV-1 infection and progressively cleared this virus. On the other hand, the lack of association between past infection and age may suggest that younger individuals are possibly engaged in higher risk behavior(s) for GBV-C acquisition, a hypothesis that warrants further investigation.

After one year, the incidence density of clearance of GBV-C was found to be of 10 cases per 100 person-years, similar to 9% observed in a larger study cohort [10], whereas among individuals susceptible to GBV-C RNA infection, the incidence density of new cases was three-fold lower. This incidence density value represents only one subject (1.6%), a lower rate than observed in a group of patients co-infected with GBV-C, hepatitis C, and HIV-1 viruses on antiretroviral therapy [71]. The factors that influence the clearance of GBV-C are not known and may depend upon the presence of sufficient numbers of CD4+ T cells [9], [72]. Our result do not support the hypothesis of antiretroviral therapy interfering with GBV-C clearance, considering that only one out of the four subjects who cleared GBV-C RNA was on treatment. Likewise, the role of E2 antibody development in GBV-C RNA clearance remains unclear. In our study, E2 antibody was not developed in the patients who cleared de GBV-C virus, verified at visit 5 (after one year of enrollment) therefore, our results may underestimate the lifetime of the GBV-C infection. Similarly the lacking of E2 antibody detection may represent first, the absence of sensitivity of the nucleic acid tests to detect low levels of viral replication by the conventional methods. The viral replication can also occur at higher or lower detection rates, as occurs with HCV in some cases. Second, longer window period between the clearance of viremia and the antibodies detection may exist. Third, the lack of specificity of the EIA for E2 antibody can lead to undetectable level of antibodies. Since our analysis was based on two time points we do not know when GBV-C was cleared and whether more time is required for antibody detection. Moreover, we still do not know if the spontaneous resolution of GBV-C viremia is necessarily followed by the appearance of E2 antibodies [72]. Therefore, it is also important to know the GBV-C genotype if we consider that difference in GBV-C strain circulating within populations might affect the progression of HIV disease [73], [74].

We also cannot rule out fluctuating GBV-C RNA levels in the four subjects with undetectable results after baseline positive viremia, which may bounce back up. A longer follow-up would necessary to assess this possibility.

At enrollment, dual HIV-1 and GBV-C viremic subjects exhibited a trend toward an inverse correlation between GBV-C and HIV-1 viral load, which was maintained after one year of follow-up if they remained off antiretroviral therapy. We hypothesized that if GBV-C exerted a beneficial effect on the immune system of the HIV infected patient, the GBV-C load should correlate with higher CD4+ T cell counts or lower HIV RNA copies/ml, reflecting inhibition of HIV replication. A negative correlation between GBV-C load and HIV-1 load as well as positive correlation between GBV-C load and CD4+ T cell counts has been demonstrated previously among chronically HIV-infected patients [10]. In our study, we found a trend towards a negative correlation between the GBV-C load and the HIV-1 load but no correlation between the GBV-C load and the number of CD4+ cells at the earlier visit. The study population may explain these findings as it is composed of recently HIV-1 infected individuals. Additionally, the median viral load of GBV-C at one year after enrollment was higher comparing to the baseline levels, but this change was not statistically significant. We cannot conclude whether the increase in GBV-C viral load is due to viral transactivation exerted by HIV-1 replication, or due to immunodeficiency progression. Nevertheless our data is consistent with a recent study showing that GBV-C viremia appears to reactivate in HIV-infected individuals, resulting in a significant increase of GBV-C viral load [21].

The results of the phylogenetic analysis identified genotypes 1, 2a, and 2b, consistent with other reports in Brazil [47], [53], [54] in which the frequency of viral isolates is similar, such as the USA, Africa, and Europe. The isolates were not clustered and the presence of genotypes 1 and 2 in Sao Paulo was not unexpected, given the high percentage of individual reporting African or European origins in this city. The extent of the GBV-C sequence diversity is limited. Consequently, little information is available for the geographical distribution of the genotypes and the influence on the clinical outcome. A previous report has shown that CD4+ T cell counts tended to be lower in patients infected with genotype 2a compared to those with genotype 2b [73]. In our study, the genotype 1 presented higher values of GBV-C viral load, followed by the genotype 2a and 2b, both at similar levels. It is difficult to conclude whether the GBV-C genotype influences the clinical outcome due to the limited number of cases in this cohort after such stratification. Larger studies, including different regions of Brazil, may demonstrate if the genotype has a stronger impact in the clinical course of HIV-1 infection or on surrogate markers of progression.

Despite the particular sampling of the cohort, predominantly MSM, and the small sample size after stratification by GBV-C genotype, we performed detailed evaluations of the prevalence and genotyping distribution in a cohort of recently HIV-1 infected subjects. This is the first report of density incidence in the GBV-C infection in this population from Southeast Brazil. Potential diverse distribution of GBV-C virus may also account for different interaction between both viruses in different regions of the world, which should be further investigated.
